# Synthesis and Characterization of Flame Retarded Rigid Polyurethane Foams with Different Types of Blowing Agents

**DOI:** 10.3390/ma16227217

**Published:** 2023-11-17

**Authors:** Marcin Zemła, Sławomir Michałowski, Aleksander Prociak

**Affiliations:** Department of Chemistry and Technology of Polymers, Cracow University of Technology, Warszawska 24, 31-155 Cracow, Poland

**Keywords:** material testing, thermal properties, mechanical properties, rigid polyurethane foams, halogen-free phosphorus flame retardant

## Abstract

In this study, rigid polyurethane foams modified with non-halogenated flame retardant were obtained. The foams were synthesized using two systems containing different blowing agents. In the first one, cyclopentane and water were used as a mixture of blowing agents, and in the second one, only water was used as a chemical blowing agent. The systems were modified with the additive phosphorus flame retardant Roflam F5. The obtained modified foams were tested for their flammability and basic properties, such as apparent density, closed-cell contents and analyses of the cell structures, thermal conductivity, mechanical properties, and water absorption. Increasing the content of Roflam F5 caused a decrease in temperature during the combustion of the material and extended the burning time. The addition of 1.0 wt.% phosphorus derived from Roflam F5 caused the modified rigid polyurethane foam to become a self-extinguishing material. The increase in the content of Roflam F5 caused a decrease in the total heat release and the maximum heat release rate during the pyrolysis combustion flow calorimetry. The foams with the highest content of flame retardant and foamed with a chemical-physical and chemical blowing agent had a lower total heat release by 19% and 11%, respectively, compared to reference foams.

## 1. Introduction

Polyurethanes (PURs) are one of the most important polymeric materials behind polyethylene, poly(vinyl chloride), polypropylene, and polystyrene. Due to the easy possibility of changing the PUR system, which mainly consists of polyol, isocyanate, catalyst, surfactants, and blowing agents, their properties can be modified [1,2]. As a result, they are used in various industries such as automotive, footwear, furniture, textiles, and construction [3,4]. This makes it one of the fastest-growing groups of polymer materials. Rigid polyurethane foams (RPURFs) are the most widely produced polymeric foam material. An advantage of low apparent density RPURFs is good mechanical strength and dimensional stability [5].

RPURFs are materials with the best thermal insulation properties among materials produced on an industrial scale [6]. This feature, combined with good mechanical properties, makes it possible to use RPURFs as thermal insulation in buildings and refrigeration industries [7,8,9]. However, due to their organic nature and large specific surface area, RPURFs are flammable [10,11]. To apply them in construction as thermal insulation and construction materials, flame retardation of polyurethane foams is necessary for safety purposes [12,13]. In order to obtain the required flammability class of materials, it is necessary to add flame retardants that will stop the material’s burning process or slow it down [14]. Flammability reduction is most commonly achieved by adding compounds dispersed in the material and not forming a chemical bond with the polymer chain [15]. Other methods include adding compounds containing reactive groups that allow bonding to the polymer chain or coating the foams with protective coatings [16].

Due to their effectiveness, organic compounds containing chlorine or bromine are popular flame retardants. During combustion, chlorine or bromine radicals are formed, inhibiting oxidation reactions by acting as radical scavengers in the gas phase [17]. Halogen-based compounds are used as additive and reactive flame retardants. Despite effective combustion inhibition, they are gradually withdrawn from use due to their toxicity and negative environmental impact [18,19]. The addition of halogen derivatives in the foam causes the emission of large amounts of dense smoke and highly toxic compounds during a fire [20,21].

In recent years, the use of halogenated flame retardants has been limited. Therefore, new solutions that are less toxic are being investigated. Combinations of already known flame retardants with other compounds are investigated, thus seeking to enhance the flame retardant effect [22]. For example, aluminum hydroxide, aluminum oxide hydrate, or magnesium hydroxide are used [23]. About 40% of the flame retardant market is estimated to be aluminum hydroxide. The addition of such compounds reduces the temperature by endothermic dehydration reactions and the process of water evaporation. Aluminum silicates such as bentonite or halloysite also have a similar principle of operation to hydroxide flame retardants [24].

Expanded graphite is also becoming more and more popular. It lowers the temperature and creates a layer on the material, separating the rest of the material and inhibiting the spread of the flame. The layer insulates the undamaged material, slowing down the further degradation of the material and also reducing the formation of dense smoke [25]. The great advantage of expanded graphite is its non-toxicity. However, it affects the decreased thermal insulation properties of the modified PUR materials [26].

Phosphorus-containing compounds are an important group of flame retardants in PUR materials. Mainly used are aryl and alkyl phosphates, phosphonates, and phosphites, as well as inorganic phosphorus compounds, such as poly(ammonium phosphate), poly(melamine phosphate), and red phosphorus [6,27]. However, the addition of inorganic solid phosphorus flame retardants usually increases the viscosity of the polyurethane system, which makes application more difficult and may disturb the foaming process [14,28]. Additionally, such types of flame retardants are not as effective as halogen flame retardants, so they should be used in larger quantities, which may affect the physical and mechanical properties of foamed materials [29]. Liquid phosphorus-based flame retardants often have a plasticizing effect on the polyurethane matrix, causing deterioration of the mechanical properties and foam dimensional instability [30]. They can be additive or reactive flame retardants. When burned, they decompose into non-volatile phosphoric acid. It creates a thin protective layer, reducing the flow of heat and oxygen [31]. In the gas phase, they work very effectively as radical scavengers [32]. They have low toxicity to the environment. It is a halogen-free solution, so nontoxic gases, such as hydrogen chloride or hydrogen bromide, are released during combustion. It has been shown that isopropylated triphenyl phosphate does not show acute toxicity, genotoxicity, or allergic reactions in humans, and its persistence in the environment is assessed at a medium level. However, they have a very high toxicity to the aquatic environment [33]. They often exhibit synergistic properties with other flame retardants, such as nitrogen or boron compounds [34]. The gaseous products of the nitrogen compound decomposition form a charred foam layer on the material’s surface, and in the gas phase, they act as free radical scavengers. Additionally, the released nitrogen dilutes the combustible gases resulting from the decomposition of the polymer [35,36].

In this study, the effect of halogen-free phosphorous flame retardant Roflam F5 on the flammability and other typical physical and mechanical properties of RPURFs was determined. Two PUR systems were foamed with (i) a chemical blowing agent, (ii) a chemical blowing agent, and cyclopentane as a physical blowing agent.

## 2. Materials and Methods

### 2.1. Materials

The following raw materials were used for the synthesis of RPURFs:

- Isocyanate: PMDI (Polymeric diphenylmethane 4,4′-diisocyanate); NCO% = 31 wt.%; average functionality = 2.7; viscosity = 200 mPa·s (25 °C); Minova Ekochem S.A (Siemianowice Śląskie, Poland).
- Polyol: Rokopol^®^ RF-551, LOH = 420 mgKOH/g; average functionality = 4.5; viscosity = 3020 mPa·s (25 °C); PCC Rokita SA (Brzeg Dolny, Poland);
- Catalyst: Polycat^®^ 9; Tris-(dimethylaminopropyl)amine; Evonik Industries AG (Essen, Germany);
- Surfactant: Niax™ silicone SR-321; Momentive Performance Materials Inc. (Niskayuna, NY, USA);
- Physical blowing agent: Cyclopentane; Brenntag Polska Sp. z o.o. (Kędzierzyn-Koźle, Poland);
- Chemical blowing agent: Carbon dioxide generated in the reaction of water and isocyanate groups;
- Flame retardant: Roflam F5; Phenol isopropylated phosphate; phosphorus content: 8.5 wt.%, viscosity = 50 mPa·s (25 °C); PCC Rokita SA.

### 2.2. Foam Preparation

RPURFs were obtained using the one-step method by mixing the polyol premix (component A) and the isocyanate (component B) with a mechanical mixer for 8 s. The polyol premix used to prepare the reference material consisted of a polyol, a surfactant, a catalyst, and a suitable blowing agent. Component A was used to synthesize modified foams containing phosphorus flame retardant (Roflam F5). The amount of added flame retardant was selected so that the phosphorus content was equal to 0.5, 1.0, and 1.5 wt.% of the appropriate element in relation to the weight of the reference foam. The reaction mixture was poured into an open mold with dimensions of 250 × 250 × 100 mm^3^, which ensured free rise and cross-linking of the foam. The materials thus obtained were aged at room temperature for 24 h.

Two systems of RPURFs were used in the research. The first system (S1) was foamed using the chemical–physical system of blowing agents consisting of water and cyclopentane. The second system (S2) was foamed with water as a chemical blowing agent (Table 1).

### 2.3. Test Method

During the synthesis of the foams, characteristic times such as gel time, rise time, and tack-free time were determined. The gel time was measured from the moment of mixing components A and B to the point at which it was possible to pull the PUR threads from the foam. The rise time was measured from the beginning of the foam rise to its completion. The tack-free time was measured from the time the ingredients were mixed until the foam surface did not stick to the glass rod.

The foams were tested in accordance with the ISO Standard test. The apparent density is ISO 845 [37], the compressive strength is ISO 844 [38], the closed cells content is ISO 4590 [39], the thermal conductivity coefficients at the average temperature of 10 °C is ISO 8301 [40], the water absorption is ISO 2896 [41], and the limiting oxygen index is ISO 4589-2 [42].

The cellular structure of the foams was examined using an optical microscope equipped with a camera and the software Aphelion (Version 3.1). The number of cells per 1 mm^2^ and the anisotropy coefficient, calculated as the ratio of the height and width of the cells, were determined. The combustion process was monitored with a thermal imaging camera. Samples with dimensions of 10 × 10 × 100 mm^3^ were burned in an apparatus to determine the oxygen index at a constant oxygen concentration of 22.2 vol%. The maximum temperature during combustion, the average temperature, and the time after which 5 cm of the sample was burnt were determined.

The combustion properties were tested by microscale combustion calorimetry according to the ASTM D7309 method A at a synthetic air mixture N_2_:O_2_ volume ratio 80:20. The mass of the samples was ca. 2 mg, whereas the heating rate was 1 °C/min.

## 3. Results and Discussion

### 3.1. Processing Times

During the preparation of each foam, the appropriate processing times were determined: gel time, rise time, and tack-free time. In the case of foaming systems in which the blowing agent was water and cyclopentane (S1), increasing the amount of phosphorous flame retardant (Roflam F5) in the foam resulted in the processing times being extended (Table 2). A particularly large increase was observed for the tack-free time, which was twice as long for the foam containing 1.5 wt.% phosphorus compared to the reference foam without flame retardant. The rise time for systems foamed with a mixture of blowing agents (S1) and chemical blowing agent (S2) with the highest modifier content was extended by 61% and 69%, respectively, compared to the reference foams. The extension of processing times was due to the reduction in the reaction mixture’s initial viscosity by adding a low-viscosity liquid flame retardant. The times of each composition obtained from system S2 were about 20–30% shorter compared to the foams obtained on the base of system S1. This is due to the higher reactivity of the water-blown system by generating more heat in the exothermic reaction of the isocyanate with water and obtaining more primary amines, which react faster with the isocyanate to form urea bonds.

### 3.2. Cellular Structure

An important aspect in the case of RPURFs is the analysis of the cellular structure, as other foam properties, such as thermal conductivity and mechanical strength, depend on it. In the case of systems foamed with a chemical–physical composition of blowing agents, the addition of phosphorous flame retardant caused a decrease in the number of cells per 1 mm^2^ and an increase in the average cross-sectional area of cells compared to the reference foam (Table 3). This may be due to the longer gel times of the modified materials and lower viscosity of the reaction mixture, which allowed for greater expansion of gases during the foaming process and easier coalescence of small cells.

The anisotropy index of the Roflam F5 modified foams decreased below 1 compared to the reference foam in the direction parallel to the rise direction, which may be due to slower foam rise and lower viscosity of the reaction mixture. Materials with the addition of phosphorous flame retardant were characterized by a similar anisotropy coefficient in the direction parallel and perpendicular to the direction of rise.

**Figure 1:**
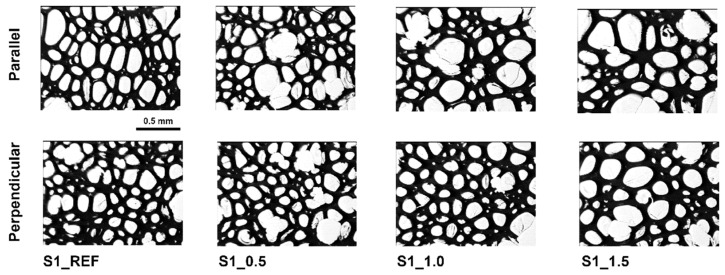
Cell structure of RPURFs with physical and chemical blowing agents.

The photos of the cell structure of foams with Roflam F5 show the lack of the characteristic cell elongation in the direction of foam rise (Figure 1). The increase in the content of the phosphorus modifier resulted in the formation of thicker cell walls.

For the water-blown foams, the addition of Roflam F5 also caused a reduction in the number of cells per 1 mm^2^ and an increased average cross-sectional area of cells compared to the reference foam (Table 4). The foams with only a chemical blowing agent had more cells per 1 mm^2^ than those blowing by cyclopentane and water, possibly due to their shorter processing times.

In the case of materials foamed with the chemical method and modified with Roflam F5, the anisotropy coefficient in the cross-section parallel to the rise direction decreased compared to the reference foam by about 17% and was close to 1.06. The anisotropy index in the cross-section perpendicular to the foam rise direction did not change significantly and was about 0.94.

**Figure 2:**
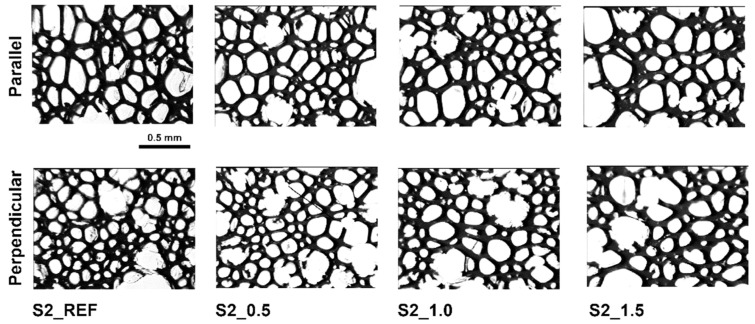
Cell structure of RPURFs obtained with chemical blowing agent.

The photos of the structures of compositions foamed with a chemical blowing agent modified with Roflam F5 show a slight elongation of the cells in the direction of rising (Figure 2) and thicker cell walls compared to unmodified foam.

The closed-cell content is an important parameter characterizing RPURFs, which affects the thermal insulation properties of the material. All obtained foam materials had a closed-cell content above 90% (Table 5). In the case of foams with a chemical–physical system of blowing agents, increasing the amount of Roflam F5 resulted in a slight decrease in the content of closed cells compared to the reference foam. This could be due to the excessively long gel time and reduced viscosity of the reaction mixture, which could result in opening cells during the foaming process.

### 3.3. Physical and Mechanical Properties

After adding phosphorous flame retardant to the water-blown system, the content of closed cells did not change significantly compared to the reference foam (Table 5). This may be due to the higher reactivity of the system compared to the system foamed with a chemical–physical blowing agent through which the reaction mixture has reached the appropriate viscosity, preventing the cells from opening.

The thermal conductivity coefficient was tested 24 h and 7 days after the foam synthesis. In the case of foams with a chemical–physical blowing agent (S1), the introduction of 0.5 wt.% phosphorus resulted in a decrease in the thermal conductivity coefficient, which may be due to the reduction in the anisotropy coefficient in the direction parallel to the foam rise direction (Table 5). The thermal conductivity of the foams with a chemical blowing agent (S2) decreased until the phosphorus content in the foam was 1 wt.%. With further increase in the amount of flame retardant in foams, thermal insulation properties decreased because apparent density increased and the content of closed cells in the obtained foam materials decreased. The heat conduction coefficient could also be influenced by the change in apparent density. As the apparent density increases, the radiation in heat transport decreases, but the conduction of the polyurethane matrix increases. For this reason, the minimum thermal conductivity coefficient for S1_0.5 and S2_1.0 can be achieved [43]. The result of testing the thermal conductivity of RPURFs with a mixture of water with cyclopentane as well as water after 7 days showed that it increased by about 5% and 17%, respectively, compared to the value measured after 24 h, which is caused by the diffusion of blowing agents from the cells and replacing them with air [44]. Inferior thermal insulation properties of materials foamed only with the chemical blowing agent are the result of a higher thermal conductivity coefficient of CO_2_ (0.0153 W/(m·K)) than that of cyclopentane (0.0114 W/(m·K)). During the examination of the thermal conductivity coefficient after 7 days, the foams that were foamed only with carbon dioxide had worse insulating properties than the material foamed with the chemical-physical system of blowing agents, which is an effect of a faster diffusion of CO_2_ than cyclopentane.

The increase in the content of phosphorous flame retardant in foams caused an increase in the apparent density of the foam materials obtained according to both systems. The foams obtained according to the S1 and S2 systems with the highest amount of modifier had higher apparent density by 14.3 and 8.5 kg/m^3^, respectively, compared to unmodified foams. The addition of Roflam F5 increased the apparent density of the foams, as a large amount of the modifier was added at the synthesis stage to ensure the right amount of phosphorus in the foam.

The compressive strength of RPURFs was tested in parallel and perpendicular directions to the foam rise due to the anisotropic nature of the obtained materials. For the composition foamed with cyclopentane–water composition, the increase in the content of Roflam F5 resulted in increased compressive strength in both measured directions due to the increase in foam apparent density (Table 5). The compressive strength of the material parallel to the foam rise direction increased by 27% and perpendicular by 76% for the content of 1.5 wt.% phosphorus in foam compared to the reference foam. In the composition with only water as a chemical blowing agent, the increase in the content of Roflam F5 resulted in increased compressive strength. The material’s mechanical parameters with the highest phosphorous flame retardant content increased in the direction parallel to the foam rise direction by 16% and in the perpendicular direction by 78% compared to the respective reference foam. The increase in compressive strength of modified foams was caused by an increase in the apparent density of the foams and by changes in the cell structure. Differences in strength resulting from the test direction decrease as the content of Roflam F5 increases, resulting from a decrease in the anisotropy coefficient in the cross-section parallel to the foam rise direction. Comparing the materials foamed with chemical–physical and chemical blowing agents, it can be stated that materials foamed only with carbon dioxide had lower mechanical strength. This may be due to a lower apparent density despite the greater number of rigid segments resulting from the formation of urea bonds.

Due to the increase in apparent density with an increase in the content of Roflam F5, the compressive strength of samples (σ_i_) with an apparent density of ρ_i_ was normalized to the apparent density of 40 kg/m^3^ according to the formula [45]:

(1) $$\sigma_{\mathrm{norm}}{=\sigma }_{i}{\left(\frac{40}{\rho_{i}}\right)}^{2.1}\;$$

**Figure 3:**
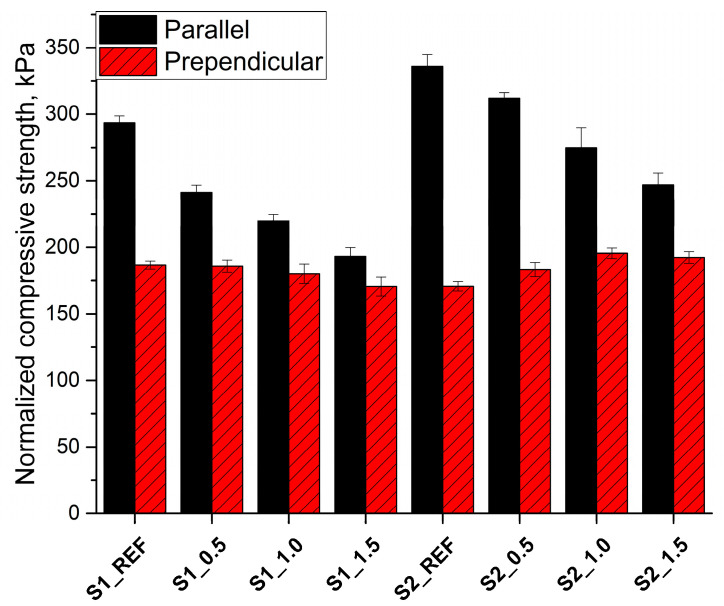
Compressive strength of RPURFs normalized to 40 kg/m^3^.

The normalization of the RPURFs’ compressive strength to one density eliminates the influence of apparent density on the mechanical properties [46]. In Figure 3, it can be seen that the normalized compressive strength in the direction parallel to the foam rise direction decreases with the increase in the Roflam F5 content for both systems. However, the normalized compressive strength of the foams obtained according to the S2 system was higher than the foams from the S1 system for the same phosphorus content in the foam materials. This may be due to the higher anisotropy coefficient in the direction parallel to the rise direction of the foams obtained according to the S2 system and also the higher content of urea bonds due to the higher water content in the S2 system compared to S1. The normalized compressive strength in the direction perpendicular to the foam rise direction was similar for both systems. Large changes in normalized and non-normalized compressive strength result from a much greater effect of apparent density on mechanical strength than other factors.

The water absorption of the modified foams obtained according to both systems decreased with the increasing content of Roflam F5 (Table 5). This may be due to the increased apparent density of the modified materials and more difficult water penetration into the foams. In both cases (S1 and S2), the materials with 1.5 wt.% phosphorus content had a lower water absorption by about 48% compared to the reference foams.

### 3.4. Limiting Oxygen Index

**Figure 4:**
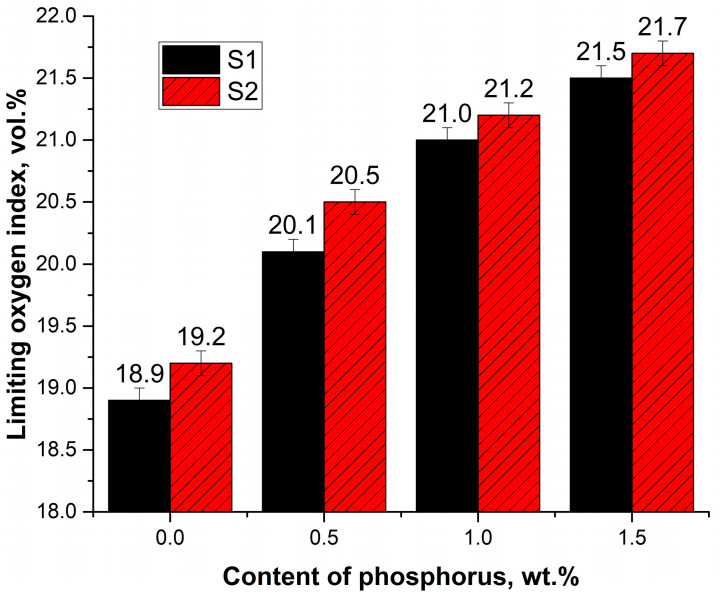
Limiting oxygen index of RPURFs.

RPURFs containing 1.0 wt.% phosphorus had a limiting oxygen index (LOI) above 21.0 vol.%, which makes it possible to classify the foams as self-extinguishing materials (Figure 4). The addition of the greatest amount of flame retardant caused an increase in the limiting oxygen index of foams with chemical–physical and chemical blowing agents to 21.5 vol.% and 21.7 vol.%, respectively. Materials foamed with water and cyclopentane, modified with the same amount of flame retardants, had lower LOI than water-blown foams, which may be because flammable gas was present in the foam cells.

### 3.5. Pyrolysis Combustion Flow Calorimetry

The method of flammability testing with the use of a pyrolysis combustion flow calorimeter consists of the pyrolysis of a few milligram samples in a nitrogen atmosphere and then complete oxidation of gaseous products resulting from the decomposition of material in a combustor at a temperature of 900 °C [47]. The foam pyrolysis takes place at a constant heating rate of 1 °C/s. The apparatus allows obtaining parameters of the combustion process such as total heat released (THR), heat release rate (HRR), heat release capacity (HRC), and temperature at the local maximum HRR (PHRR). The obtained data are presented in Table 6.

**Figure 5:**
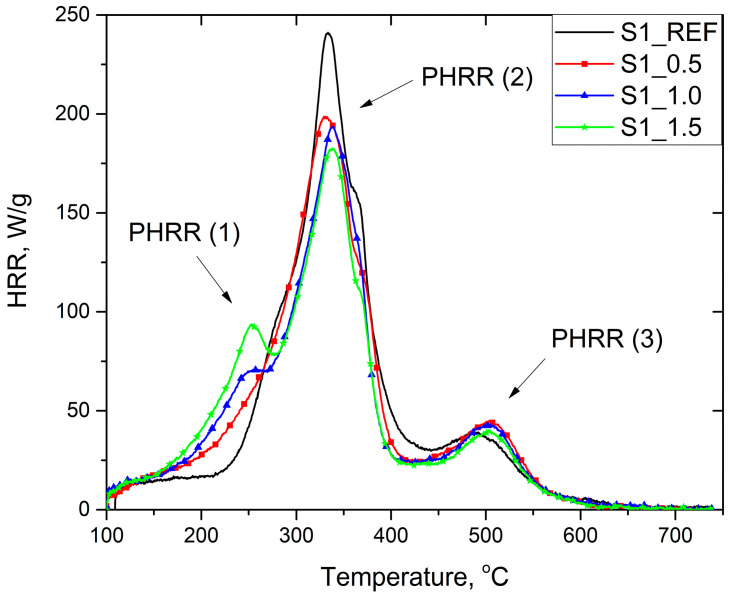
Heat release rate curves of RPURFs with physical and chemical blowing agents.

**Figure 6:**
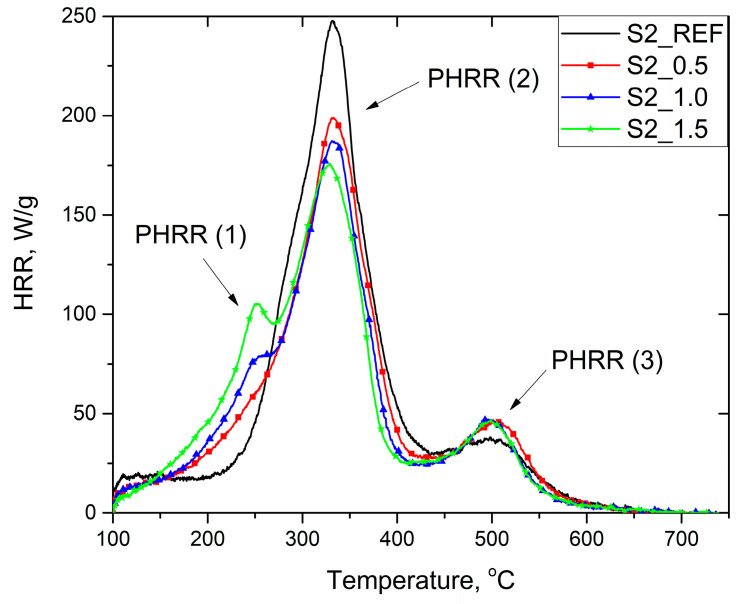
Heat release rate curves of RPURFs with chemical blowing agent.

As shown in Figure 5 and Figure 6, the thermal decomposition of unmodified RPURFs takes place in two stages. The first stage has the fastest HRR and takes place at temperatures from 230 to 440 °C. At this stage, the main decomposition of urethane bonds takes place, as well as the decomposition of the polyol [48]. The second, weaker stage takes place at a temperature of 440 to 560 °C, where aromatic compounds and char formed at the beginning of the combustion process are decomposed [13]. HRR curves of foams containing phosphorous flame retardant had another maximum at about 250 °C, which increased with increasing phosphorus content. The new peak was due to the flash point of the modifier (min. 230 °C) and thus its activation. The heat release rate of the fastest decomposition stage of PUR foams (PHRR (2)) decreased with the increase in the content of flame retardant and reached the lowest value for the S1_1.5 and S2_1.5 foams, equal to 179.5 and 184.0 W/g, respectively. The modified foams had a higher HRR in the final stage of thermal decomposition (PHRR (3)) than the reference foams, which may be due to the formation of more char in the initial stage of decomposition, which decomposes at higher temperatures.

The increase in the content of Roflam F5 resulted in a reduction in the total heat released THR in both systems. Compared to the reference foams, the S1_1.5 and S2_1.5 foams had a lower THR value by 18% and 12%, respectively. A greater reduction in the THR in the case of foams obtained according to the system foamed with chemical–physical blowing agents may result from the higher apparent density of these materials, thanks to which more protective carbon may be formed during the burning of the material [8].

Heat release capacity (HRC) describes the material’s ability to release heat during combustion. Lowering the HRC value will make the material less susceptible to ignition and reduce the heat release rate [49]. The increase in the flame retardant content causes a similar decrease in the HRC value for both systems (Table 6). A significant reduction in HRC is noticeable even with the smallest addition of Roflam F5. S1_1.5 foam had the lowest HRC, equal to 196.3 J/g·K, which decreased by about 28% compared to the reference foam.

### 3.6. Thermal Imaging Camera

The combustion process of obtained materials was observed using a thermal imaging camera in the line scanner mode, determining the maximum achieved temperature (T_max_), average temperature (T_av_), combustion time of 5 cm of the sample (t_5_), temperature peak area (S_peak_), and temperature distribution along the burning sample. The combustion of samples was performed at a constant oxygen concentration, which was 22.2 vol.%.

The presence of phosphorus flame retardant in RPURFs caused a reduction in the maximum and average temperatures during combustion compared to the material without flame retardant (Table 7). The maximum and average temperatures during the combustion process are also reduced by 62 and 29 °C, respectively. In the case of the composition of materials foamed with a chemical blowing agent, the addition of phosphorous flame retardant (S2_1.5 foams) also reduced the maximum and average temperatures compared to the values for reference foam by 111 °C and 92 °C. The time needed to burn 5 cm of the sample with the addition of a maximum amount of Roflam F5 was around 42 s in both systems, which is an extension of the combustion time by 27 s compared to the reference foam.

**Figure 7:**
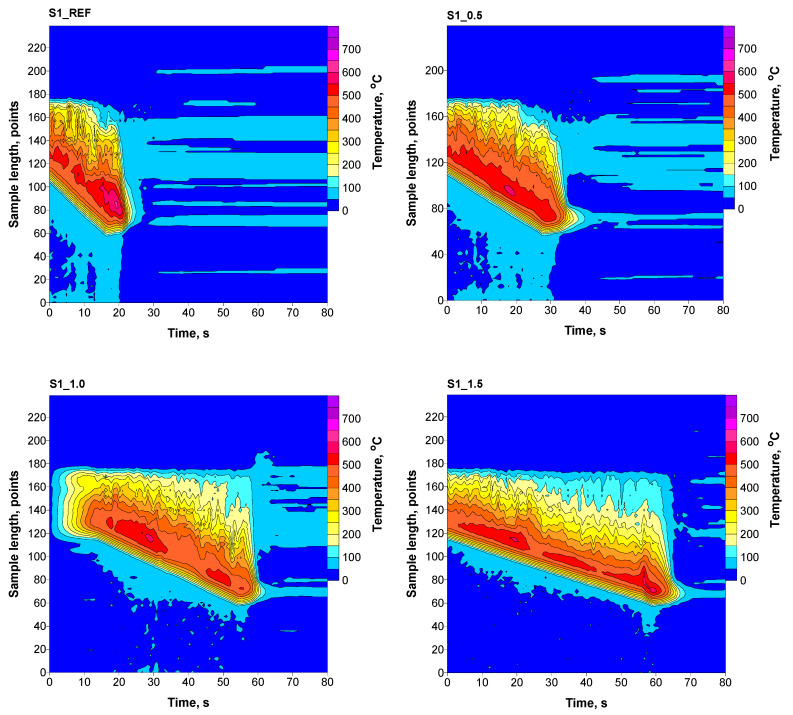
Thermograms of RPURFs with a chemical–physical composition of blowing agents.

**Figure 8:**
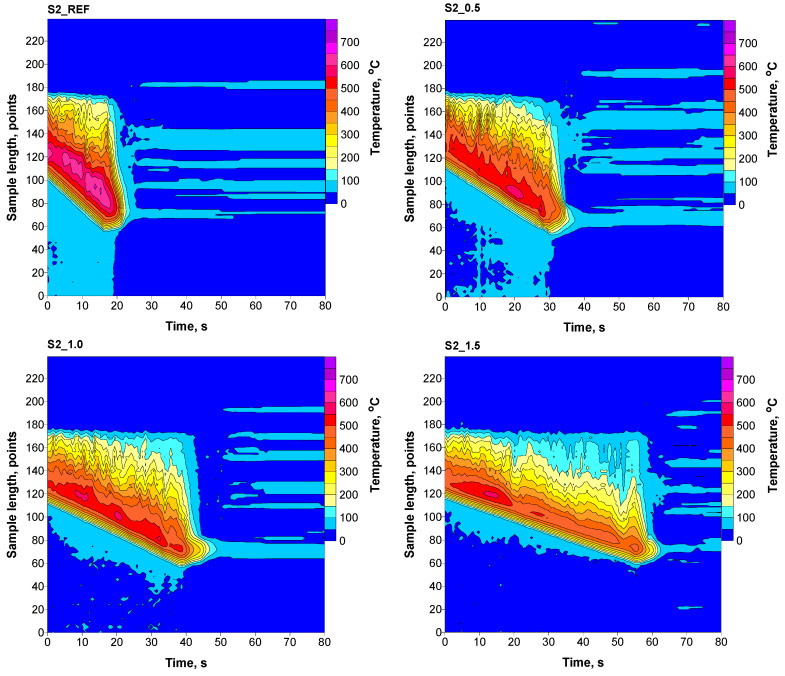
Thermograms of RPURFs with chemical blowing agent.

The area of the temperature peak depends on the amount of added Roflam F5. Increasing content of Roflam F5 causes its reduction, which may be caused by forming a protective layer, hindering the flow of oxygen and gaseous products of decomposition of the material, and acting in the gas phase as a radical scavenger. The thermograms show a significant increase in the combustion times of the sample and a decrease in the temperature of the burnt sample with an increase in flame retardant (Figure 7 and Figure 8).

## 4. Conclusions

Roflam F5 effectively reduces the flammability of rigid polyurethane foams, significantly increasing the limiting oxygen index and inhibiting the spread of flame. The content of 1.0 wt.% phosphorus in obtained materials allows us to classify them as self-extinguishing materials (21% < OI < 28%) [36].

The increase in the content of Roflam F5 caused a decrease in the total heat release, heat release capacity, and PHRR (2), the fastest degradation step of polyurethane material. The fastest stage of thermal degradation of the material has the greatest impact on the development of the combustion process; therefore, it is important to limit it. Lower values of these parameters have foams obtained with water and cyclopentane blowing agents. This may be due to the higher density of these foam materials, which allows for a more effective protective layer formation on the foam surface during a fire.

The higher content of Roflam F5 also reduces the maximum and average temperature in the flame and extends the time needed to burn 5 cm of the sample, so the modifier effectively reduces the flammability of rigid polyurethane foams.

Phosphorus flame retardant increased the apparent density of the materials, which could have been the result of adding a significant amount of modifier to the weight of the foam. This change is also reflected by an improvement in the mechanical properties of the foams.

Better thermal insulation properties of materials foamed with chemical–physical blowing agents result from a lower thermal conductivity coefficient of cyclopentane than carbon dioxide for systems foamed only with chemical blowing agents. The increase in the thermal conductivity coefficient 7 days after synthesis was higher compared to the study after 24 h for polyurethane systems with the addition of a chemical blowing agent compared to systems with a chemical-physical blowing agent composition due to the faster diffusion of carbon dioxide than cyclopentane.

## Figures and Tables

**Table 1:** Systems of RPURFs with chemical–physical and chemical blowing agents.

Component, g	S1_REF	S1_0.5	S1_1.0	S1_1.5	S2_REF	S2_0.5	S2_1.0	S2_1.5
**Polyol, RF-551**	100				100			
**Surfactant, SR-321**	2				2			
**Chemical blowing agent, Water**	2				4			
**Physical blowing agent, Cyclopentane**	5.95				-			
**Catalyst, POLYCAT 9**	1.5				1.5			
**PMDI, EKOPUR B**	I_NCO_ = 1.1				I_NCO_ = 1.1			
**Flame retardant,** **Roflam F5**	0.0 (0.0) *	18.7 (0.5) *	37.4 (1.0) *	56.1 (1.5) *	0.0 (0.0) *	20.8 (0.5) *	41.6 (1.0) *	62.5 (1.5) *

* (Phosphorus content in the foam, wt.%).

**Table 2:** Processing times for foaming PUR systems without and with phosphorus flame retardant.

Foam Symbol	Rise Time, s	Gel Time, s	Tack-Free Time, s
**S1_REF**	75 ± 3	71 ± 1	107 ± 2
**S1_0.5**	100 ± 1	78 ± 1	142 ± 3
**S1_1.0**	125 ± 1	90 ± 1	170 ± 1
**S1_1.5**	127 ± 1	106 ± 1	211 ± 1
**S2_REF**	49 ± 1	55 ± 1	89 ± 1
**S2_0.5**	66 ± 1	57 ± 1	100 ± 1
**S2_1.0**	75 ± 2	65 ± 1	135 ± 1
**S2_1.5**	80 ± 1	72 ± 1	176 ± 1

**Table 3:** Parameters of the cellular structure of RPURFs obtained with physical and chemical blowing agents.

Foam Symbol	Direction	Number of Cells/1 mm^2^	Average Cross-Sectional Area of Cells × 10^3^, mm^2^	Anisotropy Index
**S1_REF**	Parallel	32 ± 2	11.7 ± 0.6	1.29 ± 0.09
	Perpendicular	40 ± 1	9.6 ± 0.4	0.90 ± 0.02
**S1_0.5**	Parallel	24 ± 1	17.9 ± 0.4	0.93 ± 0.05
	Perpendicular	35 ± 3	11.7 ± 1.0	0.92 ± 0.07
**S1_1.0**	Parallel	26 ± 4	15.2 ± 1.9	0.93 ± 0.06
	Perpendicular	28 ± 3	13.7 ± 1.8	0.84 ± 0.03
**S1_1.5**	Parallel	20 ± 1	19.3 ± 2.0	0.88 ± 0.04
	Perpendicular	29 ± 4	11.8 ± 2.1	0.88 ± 0.04

**Table 4:** Parameters of the cellular structure of RPURFs obtained with chemical blowing agent.

Foam Symbol	Direction	Number of Cells/1 mm^2^	Average Cross-Sectional Area of Cells × 10^3^, mm^2^	Anisotropy Index
**S2_REF**	Parallel	38 ± 3	10.8 ± 1.1	1.28 ± 0.04
	Perpendicular	49 ± 2	7.1 ± 0.8	0.96 ± 0.01
**S2_0.5**	Parallel	33 ± 2	14.2 ± 0.8	1.11 ± 0.05
	Perpendicular	45 ± 5	10.9 ± 1.4	0.92 ± 0.03
**S2_1.0**	Parallel	25 ± 2	20.7 ± 3.4	1.00 ± 0.07
	Perpendicular	37 ± 5	11.2 ± 1.2	0.93 ± 0.03
**S2_1.5**	Parallel	28 ± 2	15.3 ± 1.2	1.06 ± 0.04
	Perpendicular	33 ± 4	13.8 ± 1.4	0.94 ± 0.03

**Table 5:** Physical and mechanical properties of RPURFs.

Foam Symbol	Closed Cells Content, %	Density, kg/m^3^	Water Absorption, %	Compression Strength, kPa		Thermal Conductivity Coefficient, mW/(m·K)	
				Parallel	Perpendicular	After 24 h	After 7 Days
S1_REF	94.2 ± 0.9	39.1 ± 1.2	3.8 ± 0.3	280 ± 5	178 ± 3	24.42 ± 0.10	25.72 ± 0.12
S1_0.5	93.0 ± 1.2	44.9 ± 1.0	2.5 ± 0.1	307 ± 7	237 ± 6	24.07 ± 0.15	25.36 ± 0.17
S1_1.0	92.7 ± 1.6	48.6 ± 0.3	2.0 ± 0.2	332 ± 7	272 ± 11	24.22 ± 0.07	25.53 ± 0.07
S1_1.5	91.6 ± 1.5	53.4 ± 0.2	1.8 ± 0.1	355 ± 12	313 ± 13	25.04 ± 0.01	26.71 ± 0.01
S2_REF	93.5 ± 1.2	34.6 ± 0.2	3.5 ± 0.6	248 ± 7	126 ± 3	26.20 ± 0.09	30.68 ± 0.19
S2_0.5	93.4 ± 0.6	37.0 ± 0.1	2.9 ± 0.1	265 ± 4	156 ± 5	26.11 ± 0.19	30.56 ± 0.11
S2_1.0	92.6 ± 0.7	40.1 ± 0.1	2.1 ± 0.1	277 ± 15	197 ± 4	25.95 ± 0.01	30.25 ± 0.01
S2_1.5	92.2 ± 1.4	43.1 ± 0.3	1.6 ± 0.1	288 ± 10	225 ± 5	26.31 ± 0.06	30.77 ± 0.21

**Table 6:** Data obtained after pyrolysis combustion flow calorimeter test.

Foam Symbol	THR, kJ/g	HRC, J/(g·K)	T(1), °C	PHRR (1), W/g	T(2), °C	PHRR (2), W/g	T(3), °C	PHRR (3), W/g
**S1_REF**	30.9 ± 0.8	274.5 ± 7.8	-	-	336 ± 5	247.3 ± 9.5	499 ± 4	35.8 ± 3.9
**S1_0.5**	26.9 ± 0.6	214.0 ± 5.7	-	-	334 ± 4	195.9 ± 3.4	502 ± 5	49.1 ± 5.8
**S1_1.0**	26.5 ± 0.1	211.5 ± 2.1	252 ± 3	70.6 ± 0.8	339 ± 2	194.4 ± 1.5	505 ± 2	48.0 ± 7.6
**S1_1.5**	25.3 ± 0.8	196.3 ± 9.9	258 ± 1	93.9 ± 3.0	337 ± 2	179.5 ± 9.9	503 ± 1	40.9 ± 0.4
**S2_REF**	30.7 ± 0.1	277.5 ± 9.2	-	-	333 ± 1	250.3 ± 3.5	501 ± 1	37.5 ± 0.8
**S2_0.5**	29.9 ± 0.5	224.0 ± 8.5	-	-	328 ± 6	204.0 ± 7.6	503 ± 4	48.5 ± 0.5
**S2_1.0**	27.7 ± 0.1	208.5 ± 4.9	252 ± 6	78.2 ± 0.9	333 ± 1	185.7 ± 3.2	500 ± 3	44.3 ± 3.4
**S2_1.5**	27.1 ± 0.8	202.3 ± 9.1	254 ± 8	104.7 ± 3.9	331 ± 3	184.0 ± 9.0	501 ± 3	45.0 ± 1.3

T(1)—temperature at PHRR (1), T(2)—temperature at PHRR (2), T(3)—temperature at PHRR (3).

**Table 7:** Combustion parameters of foams of series S1 and S2.

Foam Symbol	T_max_, °C	T_av_, °C	t_5_, s	S_peak_
**S1_REF**	641 ± 51	546 ± 26	15 ± 2	36,393 ± 1130
**S1_0.5**	587 ± 13	520 ± 26	25 ± 2	33,845 ± 2484
**S1_1.0**	578 ± 9	525 ± 14	30 ± 1	31,896 ± 624
**S1_1.5**	579 ± 5	517 ± 19	41 ± 3	28,152 ± 574
**S2_REF**	677 ± 35	595 ± 13	15 ± 1	37,690 ± 2141
**S2_0.5**	599 ± 4	534 ± 8	23 ± 1	33,817 ± 649
**S2_1.0**	589 ± 15	529 ± 1	31 ± 3	31,046 ± 759
**S2_1.5**	566 ± 11	503 ± 12	42 ± 2	26,211 ± 874

## Data Availability

Data are contained within the article.
